# Supplementary material for: Monovalent anion-selective membranes fabricated via in situ interfacial polymerization
Source: Nat Commun. 2025 Oct 14;16:9120. doi: 10.1038/s41467-025-64196-2 (PMC12521509; doi:10.1038/s41467-025-64196-2)
Supplement: Supplementary file 1 — Supplementary Information [file 41467_2025_64196_MOESM1_ESM.pdf]

Supplementary Information

for

Monovalent anion-selective membranes fabricated via in-situ interfacial polymerization

Noor Ul Afsar [1], Michael Holmboe [1], C. André Ohlin[1], Niaz Ali Khan [2], Liang Ge [3]*, Tongwen Xu [3], , Naser Tavajohi [1,] *

[1] *Department of Chemistry, Umeå University, 90187, Umeå, Sweden*

[2] *Interdisciplinary Research Center for Membranes and Water Security, King Fahd University of Petroleum & Minerals, Dhahran, 31261, Saudi Arabia*

[3] *State Key Laboratory of Precision and Intelligent Chemistry, School of Chemistry and Materials Science, University of Science and Technology of China, Hefei, Anhui 230026, China*

Corresponding authors E-mail: geliang@ustc.edu.cn; naser.tavajohi@umu.se

Supplementary Figures

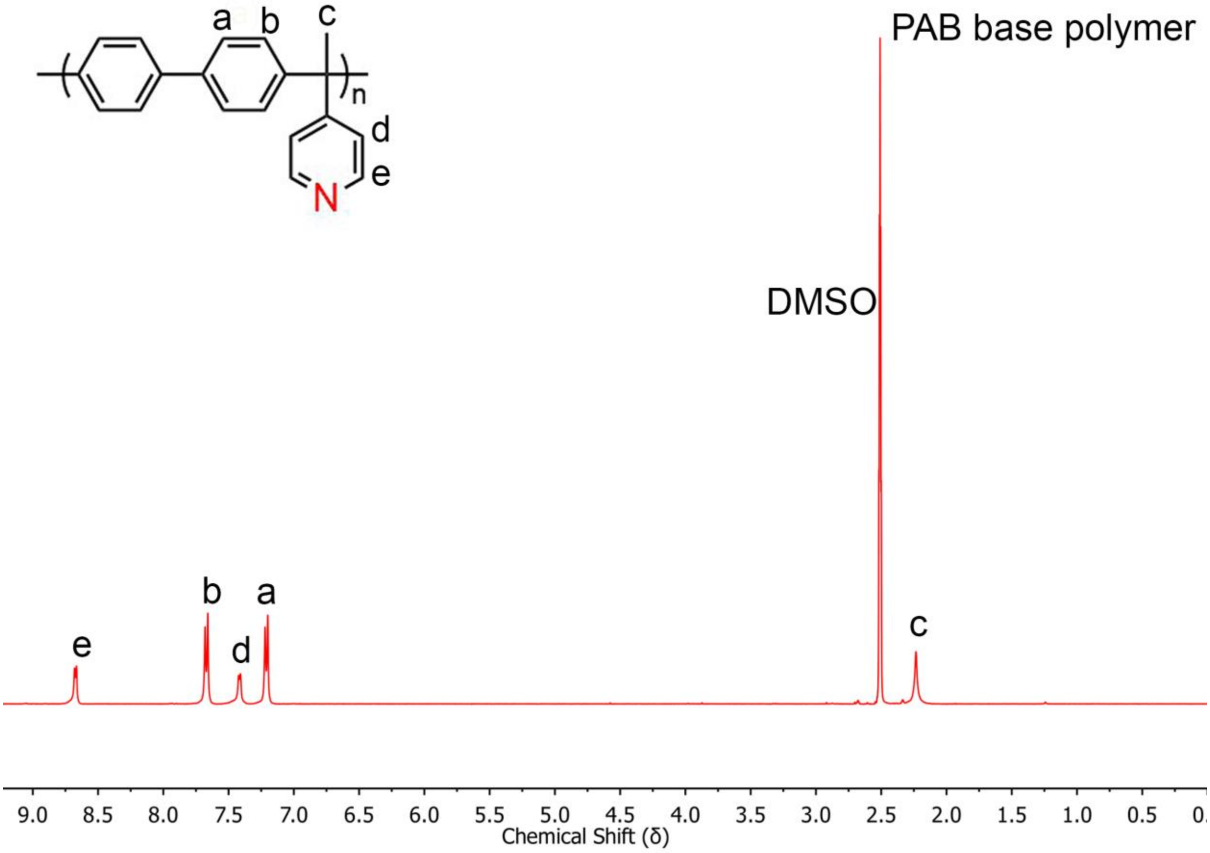

Figure S1. ^1H NMR spectrum of the poly(alkyl-biphenyl pyridine) (PAB) base polymer in DMSO-d_6 (400 MHz). The chemical shifts and assigned peaks correspond to the labelled structure of the polymer as follows: a: Aromatic protons from the para-substituted benzene ring (δ = 7.4–7.5 ppm). b: Aromatic protons from the meta-position of the central benzene ring (δ = 7.7–7.8 ppm). c: Aliphatic protons from the methylene group (δ = 2.1–2.4 ppm). d: Aromatic protons on the pyridine ring (δ = 7.3–7.5 ppm). e: Additional aromatic protons on the pyridine ring (δ = 8.6–8.8 ppm). The peak at δ = 2.5 ppm corresponds to the solvent (DMSO-d_6). The spectrum confirms the successful synthesis of the PAB base polymer with the expected structural integrity.

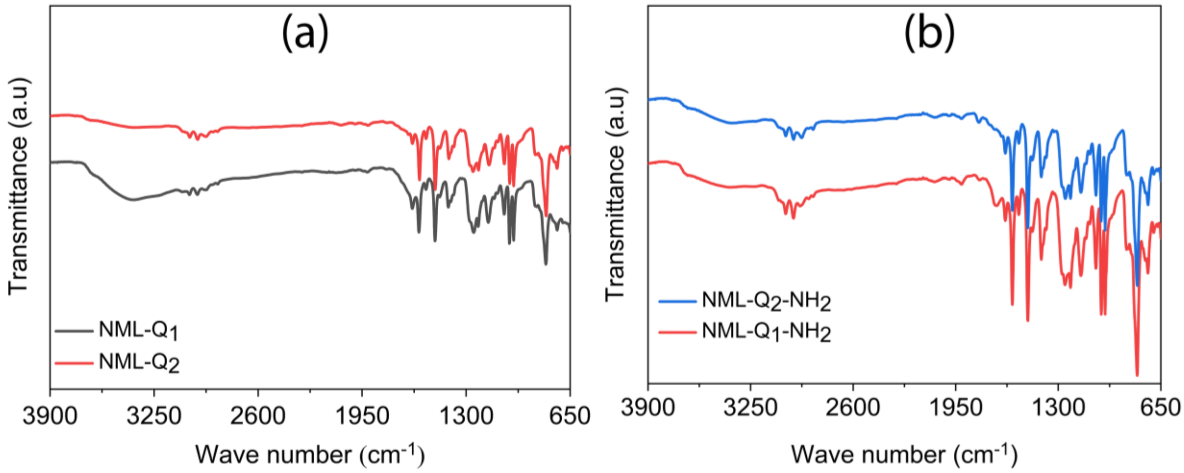

Figure S2: ATR-FTIR spectra illustrating the characteristic functional groups for: (a) NML-Q_1 and NML-Q_2, highlighting key peaks of NH_2 and $C-N^+$ (b) NML-Q_1-NH_2 and NML-Q_2-NH_2, demonstrating the changes in the spectra after functionalization with trimesoly chloride (TMC), with the appearance of new peaks corresponding to C-O and N-H groups.

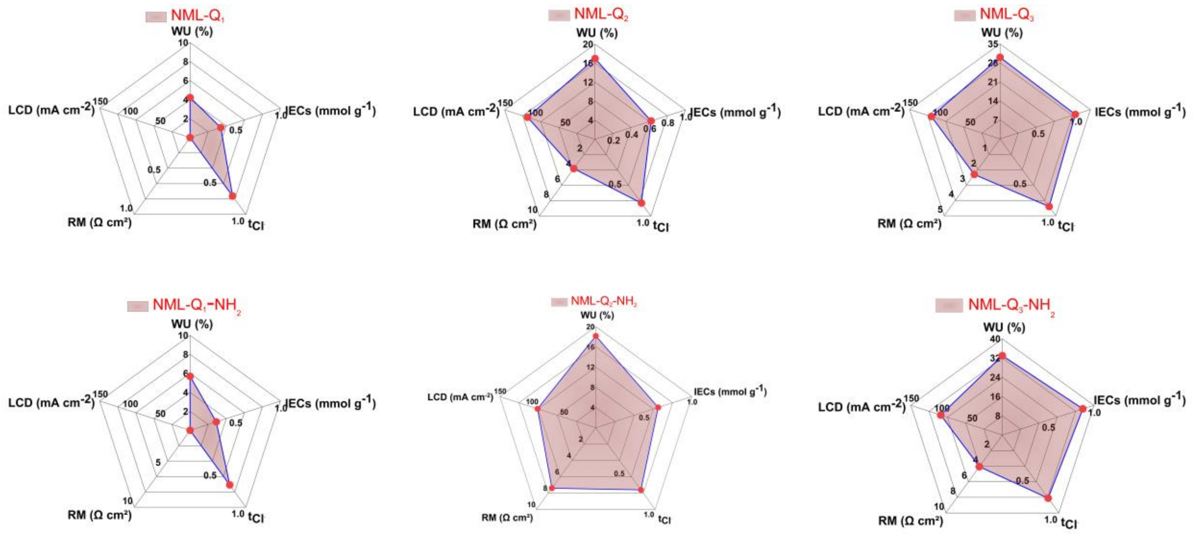

Figure S3. Radar plots depicting various physical properties of the membrane: ion exchanbe capacity (IEC), water uptatke (WU), transport number (t), membrane resistance (RM), and limiting current density (LCD). These figures provide a comprehensive overview of the membrane's performance across multiple key parameters.

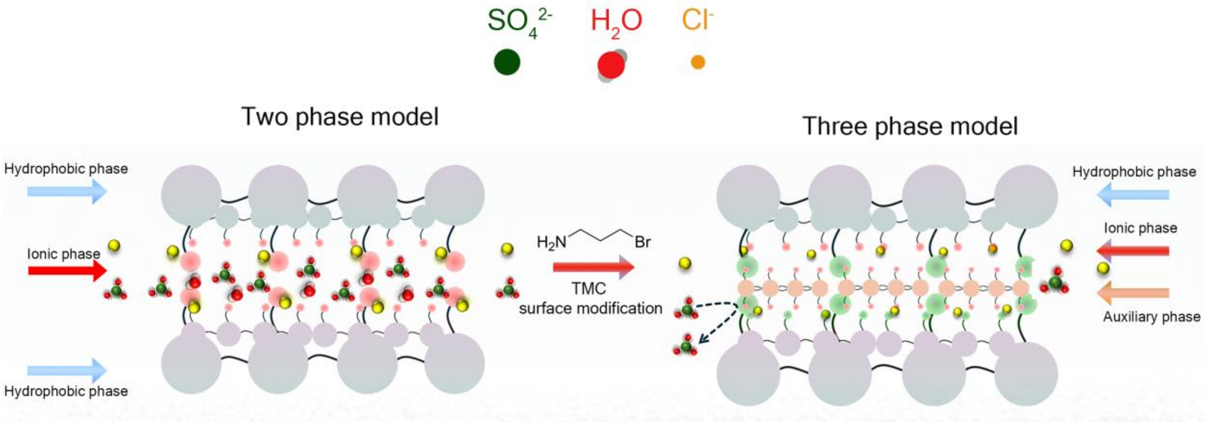

Figure S4. Two-phase and three-phase membrane structures for ion transport, showing the hydrophobic phase, ionic phase, and auxiliary phase. The hydrophobic phase provides mechanical stability, the ionic phase facilitates selective ion transport, and the auxiliary phase (present in three-phase structures) enhances transport efficiency.

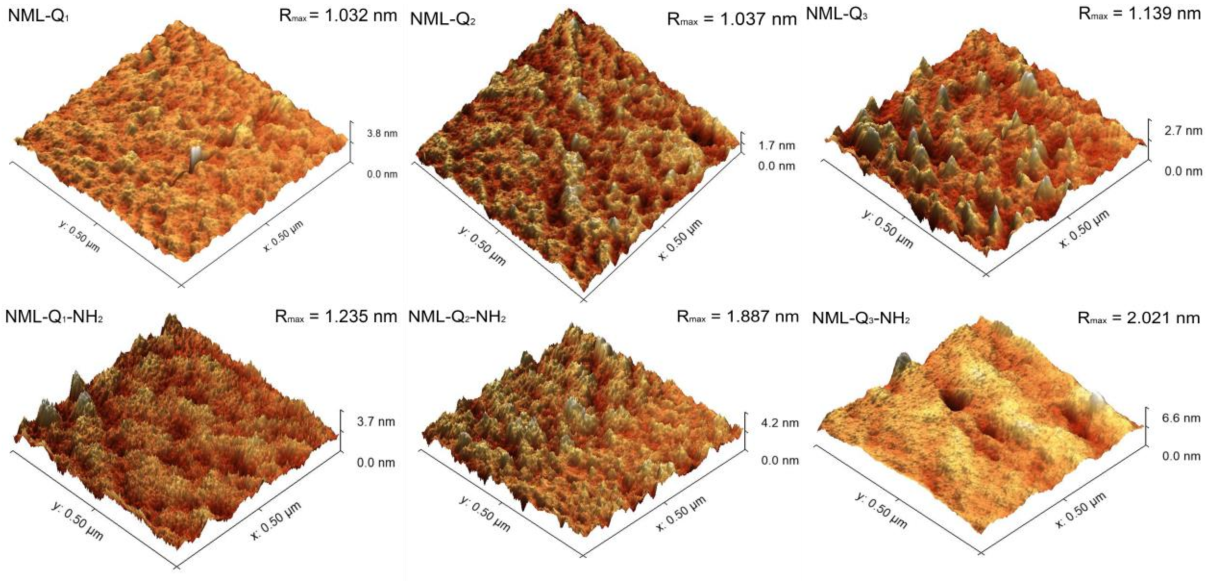

Figure S5. AFM images of NML-Q_1, NML-Q_2, and NML-Q_3 membranes before and after TMC surface modification. The roughness parameter R_{max} increases for each membrane after modification. NML-Q_1, NML-Q_2, and NML-Q_3 represent the base membranes with roughness values of 1.032 nm, 1.037 nm, and 1.139 nm, respectively. After surface modification with TMC, the roughness values for NML-Q_1-NH_2, NML-Q_2-NH_2, and NML-Q_3-NH_2 are 1.235 nm, 1.887 nm, and 2.021 nm, respectively. This increase in roughness indicates the formation of a denser and amine-functionalized layer on the membrane surface.

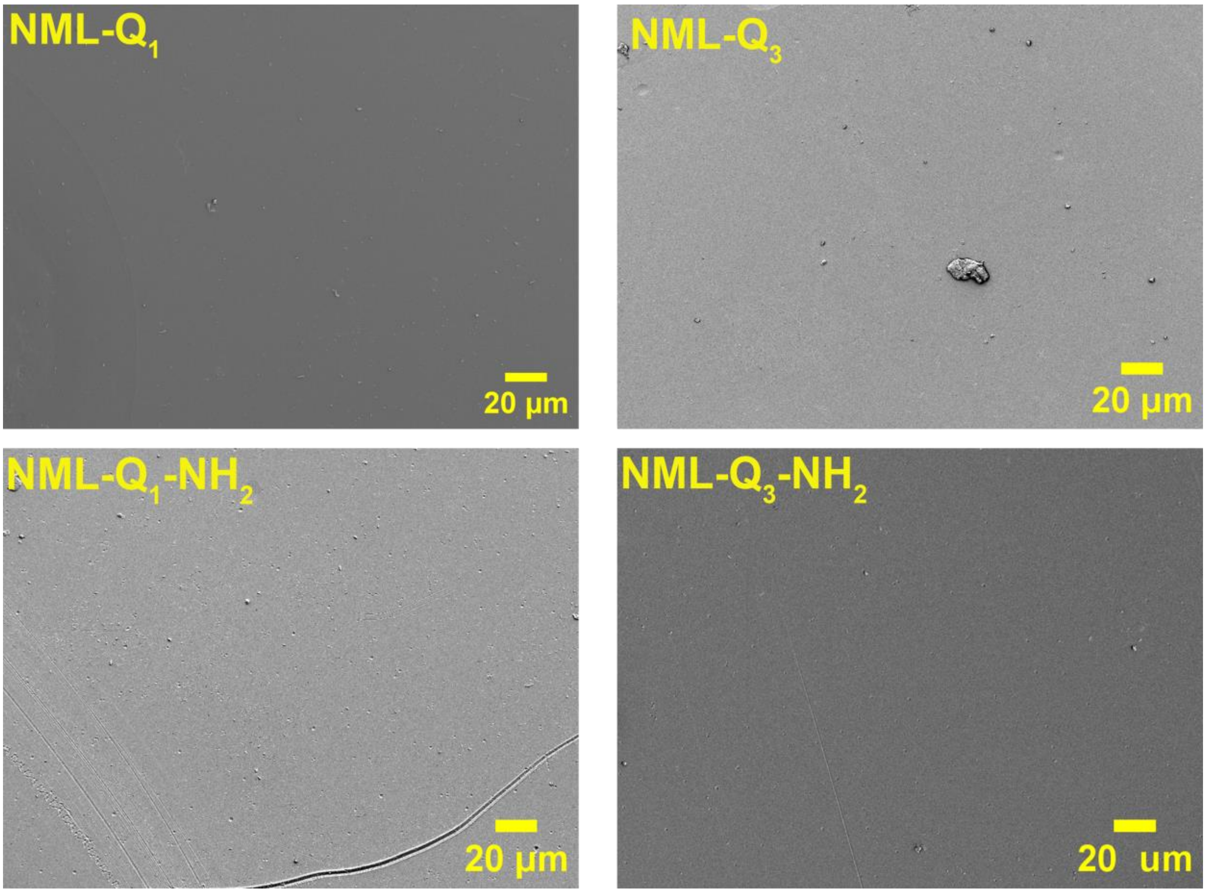

Figure S6. SEM images of NML-Q_1, NML-Q_2, and NML-Q_3 membranes before and after TMC surface modification. The surfaces of NML-Q_1, NML-Q_2, and NML-Q_3 appear smooth and exhibit uniform morphology with no visible defects. Minor agglomerations are observed on the surfaces, possibly due to the membrane fabrication process. After surface modification with TMC, the membranes NML-Q_1-NH_2, NML-Q_2-NH_2, and NML-Q_3-NH_2 maintain their smooth and uniform structure. Small surface irregularities are present but remain minimal, reflecting consistency in the modification process. The images confirm that the overall surface morphology is retained, with only slight variations observed due to the modification. Scale bars represent 20 µm for all images.

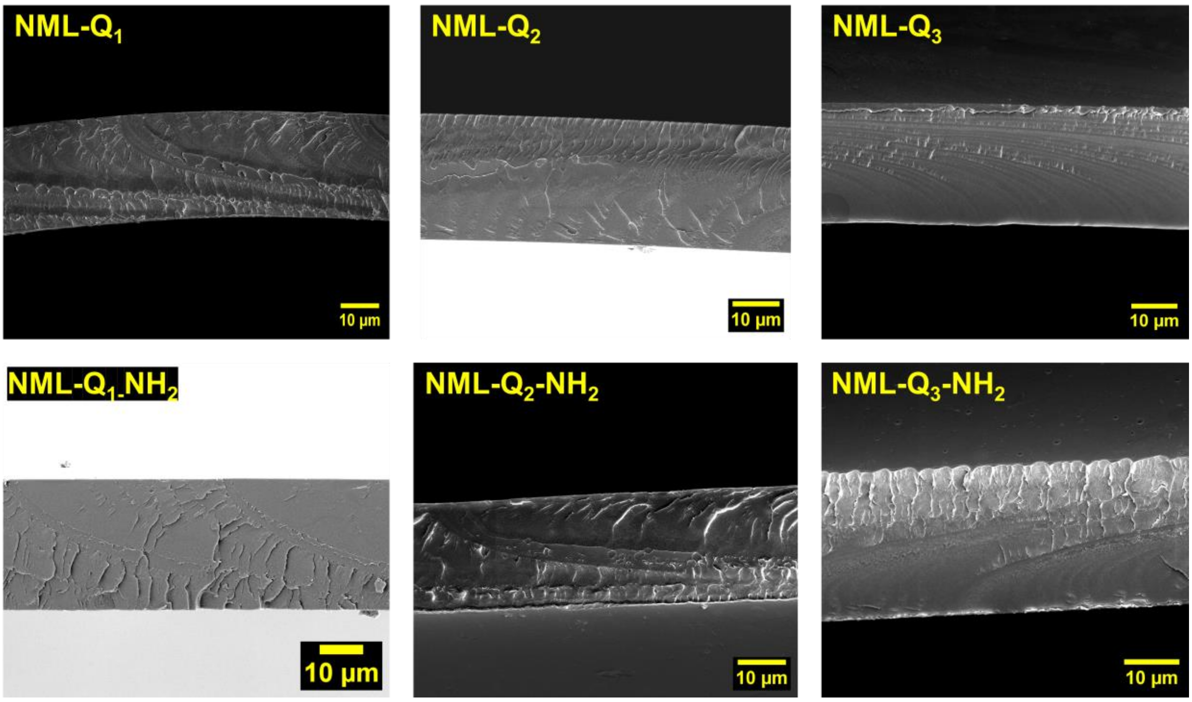

Figure S7. Cross-sectional SEM images of NML-Q_1, NML-Q_2, and NML-Q_3 samples before (top row) and after surface modification with TMC, shown as NML-Q_1-NH_2, NML-Q_2-NH_2, and NML-Q_3-NH_2 (bottom row).

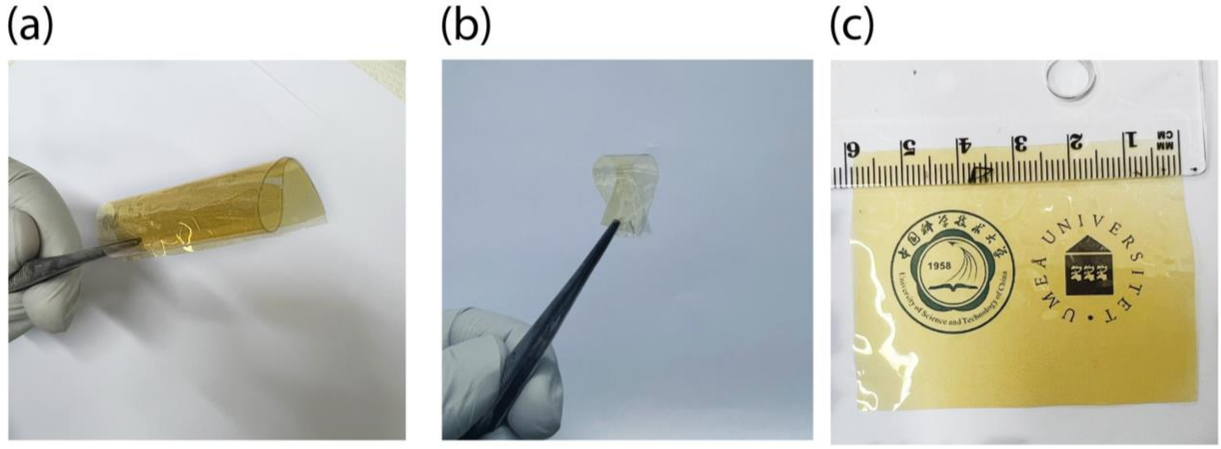

Figure S8. Physical images of the membranes demonstrating excellent flexibility (a), even at low WU, as evidenced by their ability to bend without damage (b). The images also highlight the membranes' transparency (c).

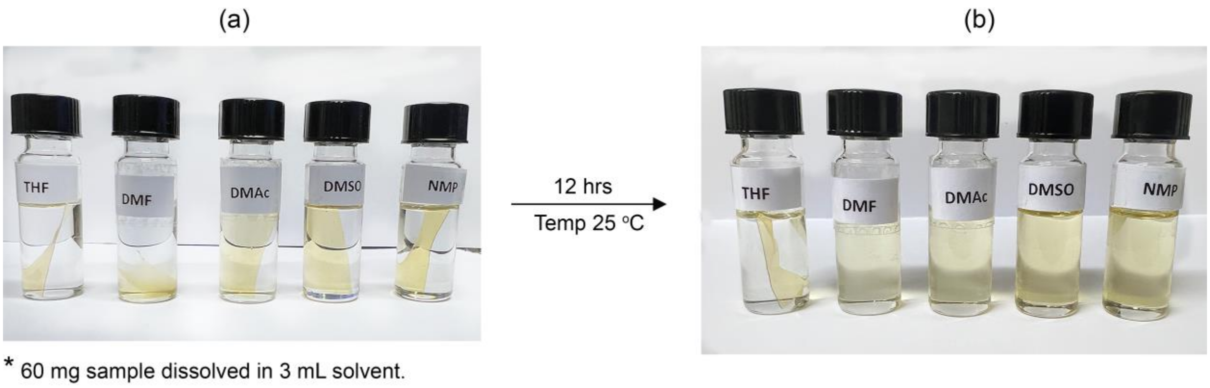

Figure S9. Solubility test of NML-Q_2-NH_2 membrane in various solvents. The membrane is soluble in DMF, DMAc, NMP, and DMSO, but not in THF. Figure 9a and 9b depict the before and after solubility test results, highlighting the membrane's solvent compatibility for ionomer solutions.

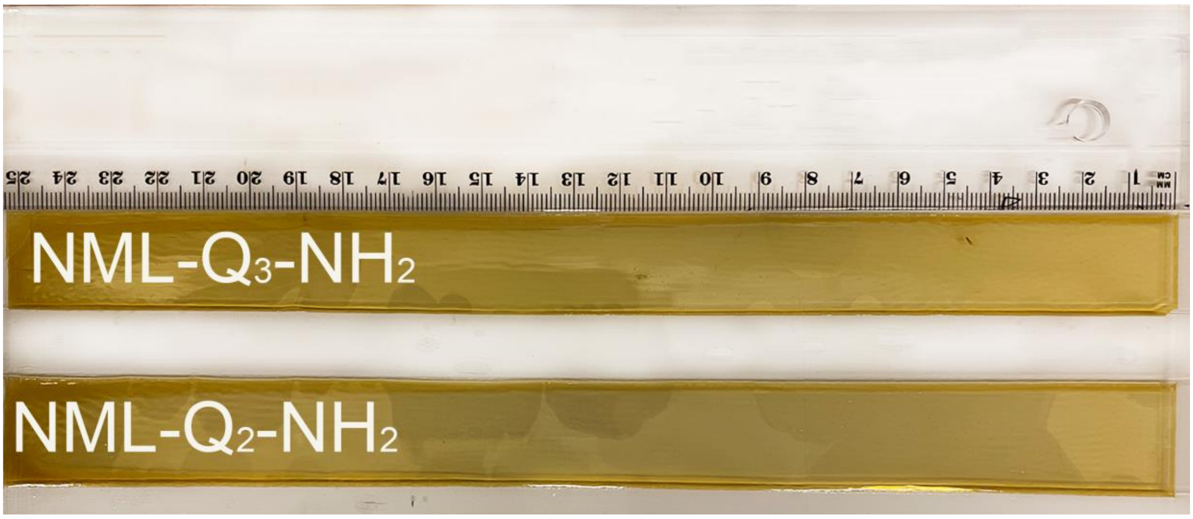

Figure S10. High-performance NML-Q_2-NH_2 and NML-Q_3-NH_2 membranes, with smooth, uniform surfaces and easy detachment from glass substrates using a water bath. Scalable for large applications, each membrane measures 25 cm in length and 2 cm in width.

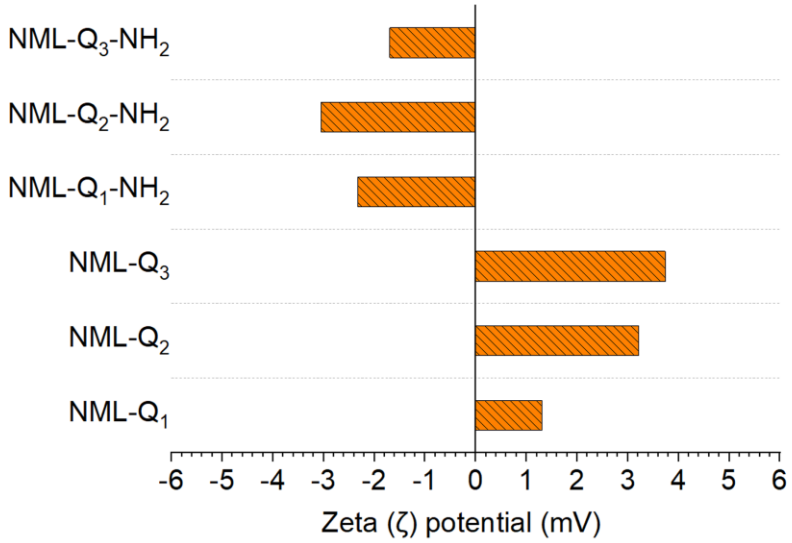

Figure S11. Zeta (ζ) potential measurements at pH 7.1 demonstrate an increase in surface charge from +1.3 mV for the NML-Q_1 membrane to +3.74 mV for the NML-Q_3 membrane, highlighting the impact of surface modifications on the membrane's charge properties.

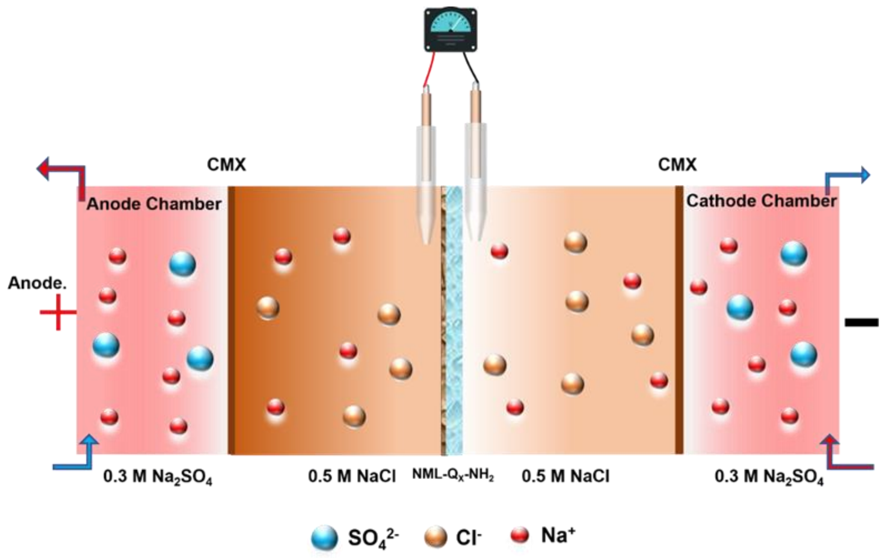

Figure S12. illustrates a schematic representation of the current-voltage (I-V) measurement setup used to evaluate the membrane resistance. The configuration includes a working electrode, a counter electrode to complete the circuit, and a ref. electrode for precise voltage measurement. These components are connected to a potentiate, which applies controlled current and record voltage across the membrane. The experiment was conducted with 0.3M Na_2SO_4 as the electrolyte solution, separated by cation exchange membranes (CEM) on either side, alongside 0.5M NaCl solution.

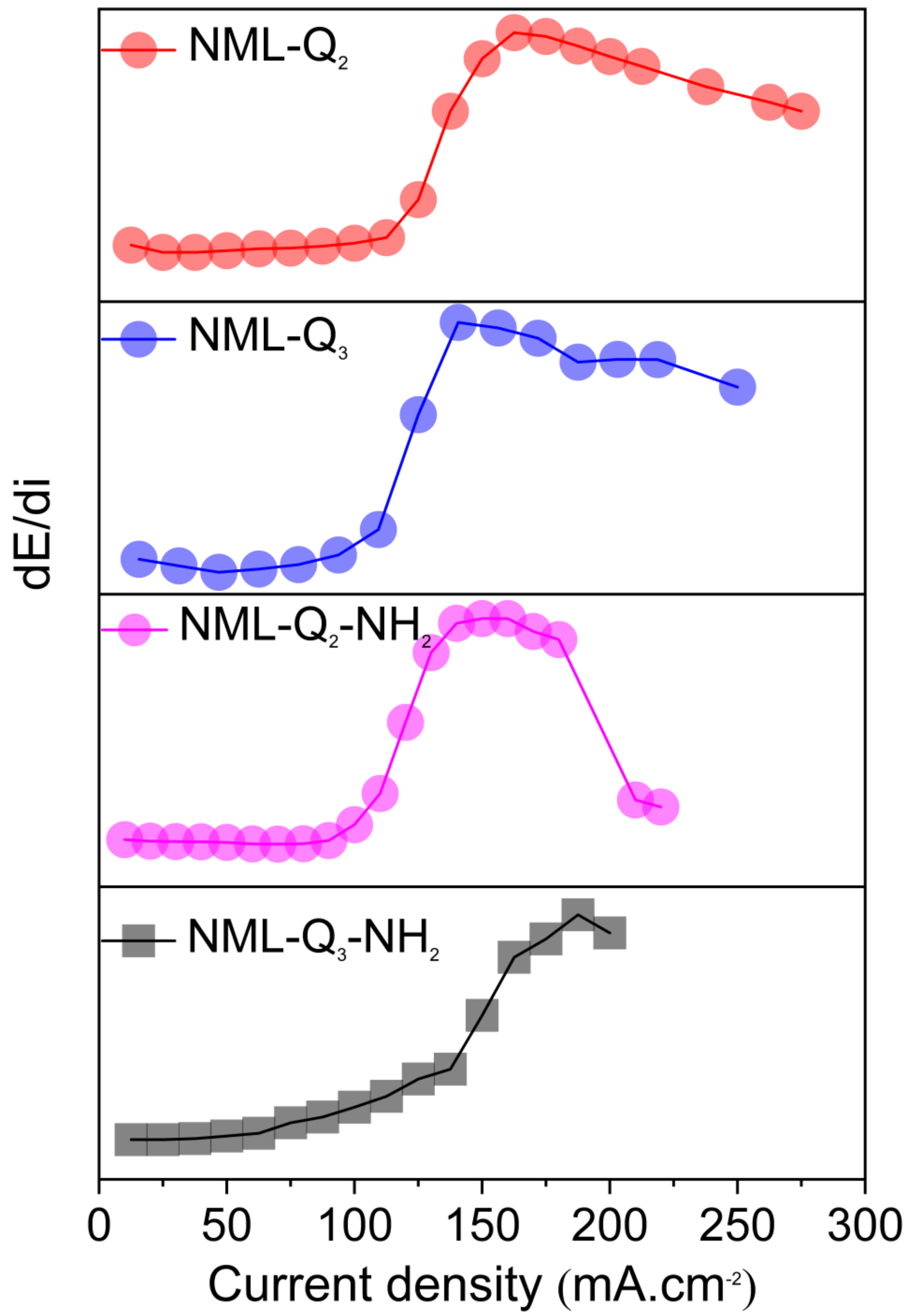

Figure S13. Current-Voltage (I-V) curve for membrane performance with corresponding dE/di values, using 0.3M Na_2SO_4 as the electrolyte, and 0.5M NaCl as the feed and receiving solutions.

Note S1. Molecular dynamics (MD) simulations.

Molecular dynamics simulations were also performed to probe the interactions between the negatively charged Cl^- and SO_4^{2-} ions with the water saturated polymer on the molecular pore scale. This was achieved by constructing a nearly cubical 3D polymer structure cross-linked 4 and 8 times in the X and Y directions (Figure S14) from deprotonated monomers.

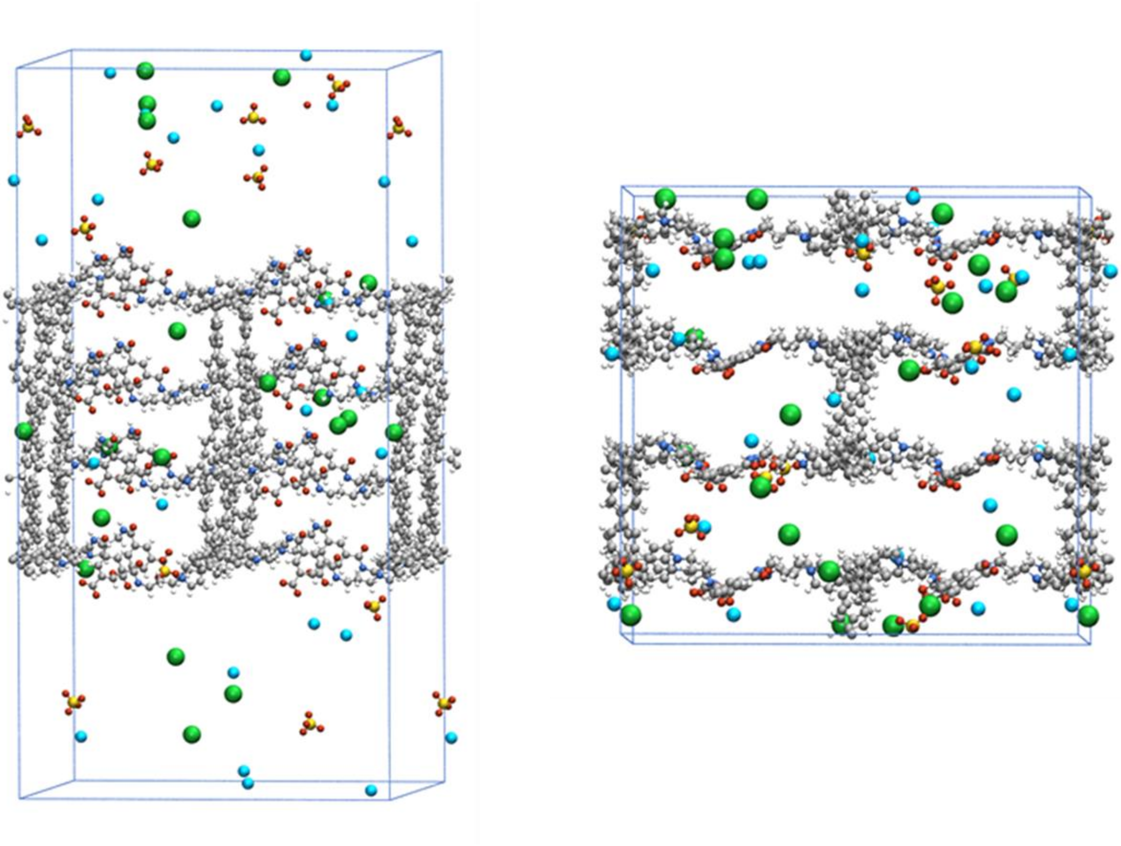

Figure S14. Initial configuration of the bulk pore system of the polymer crosslinked in X and Y directions used in MD simulations, showing randomly placed Na^+ (turquoise), Cl^- (green) and SO_4^{2-} (yellow/red) ions. Left image show y/z plane and the right image the top x/y plane of the system, respectively, with the 6500 water molecules hidden for clarity.

In total, the polymer structure contained 1732 atoms with a total charge of +16 due to the deprotonated -COOH groups. Two types of simulation cells were used, a cubical simulation cell initially extending 4.7 nm in all directions, saturated with 2800 water molecules, as well as a second system extended to 9.2 nm in the Z-direction saturated with 6500 water molecules, resulting in a bulk water pore above and below the polymer representative of the polymer/solution interface. The first type of system was used in three different simulations, containing either i) Cl^- ions, ii) SO_4^{2-} ions or iii) both Cl^- and SO_4^{2-}. The bulk water pore system was simulated with both Cl^- and SO_4^{2-} ions present. A small excess of the resp. Na^+ salt corresponding to 0.08M was used in all systems to improve sampling. To assess the effect of the polymer on the ionic solutes, equivalent simulations without the polymer were also made

for comparison. Each system was energy minimized and pressure equilibrated independently in the XYZ directions, prior a 200 ns production run in the NVT ensemble using a 2 fs timestep in Gromacs [1]. Forcefield parameters for the polymer, Na^+ and Cl^- as well as the TIP3P water model from the general CHARMM forcefield was obtained from the CGenFF server [2, 3], which was also used to construct the cross-linked polymer topology, by adapting atomtypes and topologies from the monomer and bridging moieties. Lennard-Jones parameters for SO_4^{2-} was taken from Martines-Seara and Biriukov et al., [4] whereas partial charges for both the polymer and sulphate atomtypes were adapted from electrostatic potential fitting using the RESP-A1 mode in the PyRed server [5, 6].The interactions of Cl^- and SO_4^{2-} with the polymer were determined by analysing the decrease in diffusion coefficients (Table S6) between systems with and without the polymer, as well as by computing radial distribution functions between the anionic solutes to different atomic sites in the polymer. The partitioning of Cl^- and SO_4^{2-} and their affinities to the polymer was demonstrated from Boltzmann weighted density profiles of Cl^- and SO_4^{2-} across the bulk pore-polymer.

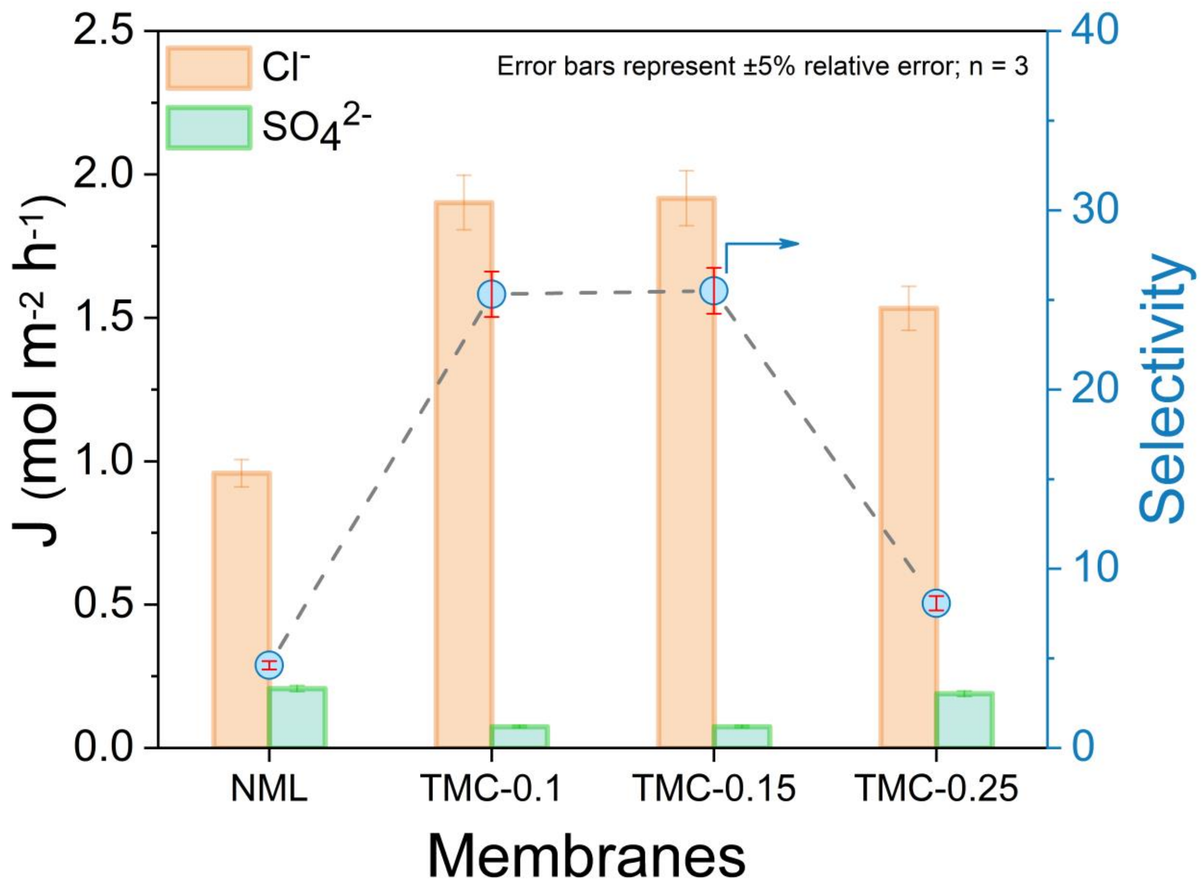

Figure S15. Ion flux and selectivity of membranes fabricated using varying concentrations of trimesoyl chloride (TMC): 0.10%, 0.15%, and 0.25% wt. The flux for monovalent anions (Cl^-) and multivalent anions (SO_4^{2-}) was determined under consistent ED conditions using a mixed salt solution containing NaCl, and Na_2SO_4. Selectivity for monovalent ions over multivalent ions was assessed to evaluate the impact of TMC concentration on membrane performance.

Supplementary Tables

Table S1. Quaternization conditions for PAB polymer membranes with varying the amount of amine, PAB, reaction temperature, and time.

S. No	Amine (mmol)	PAB (mmol)	Temp (^oC)	Time (h)
NML-Q_1	0.55	2.15	70 ^oC	12
NML-Q_2	1.10	2.15	70 ^oC	12
NML-Q_3	1.65	2.15	70 ^oC	12

Table S2. Water uptake (WU), ion exchange capacity (IEC), transport number, membrane resistance (R_m), limiting current density (LCD), and thickness for different membranes

Membrane	WU (%)	IEC (mmol g⁻¹)	Transport Number	R_m (Ω cm²)	LCD (mA cm⁻²)	Thickness (μm)
NML-Q_1	4.21 ± 0.21	0.34 ± 0.02	0.76 ± 0.04	–	–	24 ± 1.20
NML-Q_2	16.96 ± 0.85	0.62 ± 0.03	0.83 ± 0.04	3.82 ± 0.19	112 ± 6	26 ± 1.30
NML-Q_3	30.04 ± 1.50	1.00 ± 0.05	0.88 ± 0.04	2.31 ± 0.12	114 ± 6	26 ± 1.34
NML-Q_1-NH_2	5.68 ± 0.28	0.29 ± 0.01	0.71 ± 0.04	–	–	29 ± 1.45
NML-Q_2-NH_2	18.17 ± 0.91	0.65 ± 0.03	0.76 ± 0.04	7.4 ± 0.37	91 ± 5	23 ± 1.45
NML-Q_3-NH_2	32.98 ± 1.65	0.88 ± 0.04	0.81 ± 0.04	4.06 ± 0.20	101 ± 5	26 ± 1.35

Table S3. Elemental composition and XPS peak splitting of the unmodified membranes, along with the identification and description of the functional group linkages.

Element	NML-Q_1			NML-Q_2			NML-Q_3			Description
	eV	FWHM	%	eV	FWHM	%	eV	FWHM	%	
C 1*s*	284.7	0.95	61.54	284.7	1.05	67.67	284.7	0.95	58.63	C-(C, H), sp^2
	285.5	0.95	6.49	285.7	1.05	7.07	285.6	0.85	10.78	C-(C, H) sp^2 tail
	286.4	1.2	7.14	286.7	1.1	4.35	286.4	1.35	7.72	C-O, C-N
	288.0	1.1	2.39	287.9	0.9	1.13	-	-	-	C=O, N-C-N
	289.0	0.8	1.58	289.0	0.9	1.73	288.9	0.85	0.66	COOH
O 1*s*	530.9	1.25	0.61	531.0	1.1	1.19	531.0	1.2	0.50	C-O-C-N
	532.0	1.25	10.08	532.0	1.15	7.98	532.0	1.25	6.86	C=O, O=C-OH
	533.6	1.3	2.51	533.5	1.5	3.02	533.5	1.4	1.96	C-OH aromatic, O-C-O aromatic
N 1*s*	398.8	1.15	0.36	399.0	1.0	0.48	399.0	1.0	1.58	C-N (PEI)
	399.9	1.2	2.49	399.9	1.25	1.63	400.1	1.05	0.59	C-N

Element	NML-Q_1			NML-Q_2			NML-Q_3			Description
	401.7	1.5	0.53	401.8	1.3	0.55	402.0	1.3	1.09	Protonated N

Table S4. Elemental composition and XPS peak splitting of the modified membranes, along with the identification and description of the functional group linkages.

Element	NML-Q_1-NH_2			NML-Q_2-NH_2			NML-Q_3-NH_2			Description
	eV	FWHM	%	eV	FWHM	%	eV	FWHM	%	
C1*s*	284.7	1.05	57.36	284.7	1.0	59.75	284.7	1.0	58.88	C- (C, H),
	285.7	1.1	10.19	285.6	0.95	4.41	285.8	0.95	9.91	C-(C, H) sp^2 tail
	286.6	1.1	5.45	286.3	1.3	9.19	286.7	1.25	6.83	C-O, C-N
	287.9	0.95	0.94	287.9	0.9	2.68	288.1	1.2	0.40	C=O, N-C-N
	289.0	0.85	1.39	288.9	0.95	2.58	-	-	-	COOH
O1*s*	530.7	1.05	0.59	-	-	-	530.5	1.15	0.21	C-O-C-N
	532.1	1.3	12.44	532.0	1.45	10.99	532.1	1.25	9.49	C=O, O=C-OH
	533.5	1.35	2.92	533.6	1.35	3.20	533.4	1.5	2.18	C-OH aromatic, O-C-O aromatic
N1*s*	399.1	1.15	0.74	398.7	1.2	0.20	399.1	1.05	1.15	C-N (PEI)
	400.1	1.35	1.21	399.9	1.25	3.09	400.3	1.2	0.50	C-N
	401.9	1.3	0.59	401.8	1.3	0.35	402.0	1.55	1.09	Protonated N

Table S5. presents the solubility test results for NML-Q_2-NH_2 in various solvents. A 60 mg sample of NML-Q_2-NH_2 was dissolved in 3 mL of each solvent, and the samples were evaluated after 12 hrs.

Solvent	Solubility in NML-Q_2-NH_2 Membrane
DMF	✓
DMAc	✓
NMP	✓
DMSO	✓
THF	✗

The marks (✓) indicate soluble and (✗) indicate insoluble, respectively.

Table S6. represents the Ionic diameter, hydrated diameter, and hydration energy of anions.

Ion	Ionic Diameter (nm)	Hydrated Diameter (nm)	Hydration Energy (kJ mol^{-1})	Ref.
F^-	0.119	0.35	580	7
Cl^-	0.181	0.33	355	8
NO_3^-	0.189	0.34	328	7
Br^-	0.196	0.33	358	7
SO_4^{2-}	0.29	0.379	1145	7

Table S7. Swelling ratio (SR%), and water contact angle (°) of NML-Q_x membranes

Sample	Swelling Ratio (%)	Contact Angle (°)
NML-Q_1	1.7	75.69
NML-Q_2	5.6	72.42
NML-Q_3	9.8	63.42

Table S8. Chloride ion flux, moles transported, and current efficiency for different membrane samples (NML, NML-Q_1, NML-Q_2, NML-Q_3, NML-Q_1-NH_2, NML-Q_2-NH_2, and NML-Q_3-NH_2) during ED.

Sample	Cl^- ion flux (mol m^{-2} h^{-1})	Moles Transported	Current Efficiency (%)
NML	0.9584	0.000678	50.47
NML-Q_1	1.05745	0.0007475	55.67
NML-Q_2	1.34739	0.0009529	70.94
NML-Q_3	1.80489	0.001275	94.89
NML-Q_1-NH_2	2.09373	0.001480	110.20
NML-Q_2-NH_2	1.98314	0.001402	104.40
NML-Q_3-NH_2	2.00231	0.001415	105.35

Table S9. Energy consumption (kWh mol^{-1}) of ion for a mix solution (0.1 M $NaCl/Na_2SO_4/NaNO_3/NaF/NaBr$) using NML-$Q_x$-$NH_2$.

Ion	NML-Q_1-NH_2	NML-Q_2-NH_2	NML-Q_3-NH_2
Cl^-	1.36	1.08	0.57
SO_4^{2-}	12.72	63.61	15.93
Br^-	1.00	1.08	1.08
NO_3^-	0.76	0.74	0.94
F^-	4.76	5.31	4.90

Table S10. Diffusion coefficients obtained from the MD simulations of the polymer, polymer/water pore as well as polymer free systems. Due to the well-known high D value of the TIP3P water model show in the last column, D values normalized to experimental water is also shown.

	Polymer Na^+/Cl^-			Polymer Na^+/SO_4^{2-}			Polymer $Na^+/Cl^-/SO_4^{2-}$			
	Na^+	Cl^-	Water	Na^+	SO_4^{2-}	Water	Na^+	Cl^-	SO_4^{2-}	Water
***D* [m²/s]**	0.821	1.332	3.033	0.758	0.414	2.982	0.782	1.526	0.432	3.023
**err. est.**	0.030	0.052	0.003	0.003	0.034	0.016	0.015	0.080	0.057	0.006
**Norm. *D***	0.387	0.628	1.431	0.358	0.195	1.407	0.369	0.720	0.204	1.426
**Norm err.**	0.014	0.025	0.002	0.002	0.016	0.007	0.007	0.038	0.027	0.003

	Polymer+Bulk pore Na^+/Cl^-				Polymer+Bulk pore Na^+/SO_4^{2-}			Polymer+Bulk pore $Na^+/Cl^-/SO_4^{2-}$			Bulk water
	Na^+	Cl^-	SO_4^{2-}	Water	Na^+	Cl^-	Water	Na^+	SO_4^{2-}	Water	Water
***D* [m²/s]**	1.361	2.022	0.913	4.010	2.014	2.721	4.594	1.912	1.424	4.520	4.876
**err. est.**	0.090	0.006	0.022	0.008	0.002	0.018	0.037	0.094	0.060	0.005	0.054
**Norm. *D***	0.642	0.954	0.431	1.891	0.950	1.283	2.167	0.902	0.672	2.132	2.300
**Norm err.**	0.042	0.003	0.011	0.004	0.001	0.008	0.017	0.044	0.028	0.002	0.025

Table S11. Normalized ion flux and selectivity for different membranes in various ion systems. This table presents the normalized flux (mol m^{-2} h^{-1}) for different membranes in systems involving monovalent anions (Cl^-, SO_4^{2-}, NO_3^-, F^-) for the separation of divalent anions (SO_4^{2-}) based on recent studies. The P (Cl^-/SO_4^{2-}) values indicate the selectivity of specific ion systems, with the highest selectivity observed in membranes prepared *via* the ISIP process (NML-Q_2-NH_2).

Membranes	Current density ($mA \cdot cm^{-2}$)	Feed Concentration	Ion System	Flux (mol m^{-2} h^{-1})	(Normalized flux)	P (Cl^{-}/SO_4^{2-})	Ref
PAES-UIO-66Pyr	2.5	0.05 M $NaCl/Na_2SO_4$ (or 0.05 M $Na_2SO_4/NaNO_3$)	Cl^{-}/SO_4^{2-}	0.504	0.145	54.26	9
PAES-UIO-66Pyr			NO_3^{-}/SO_4^{2-}	0.7884	0.227	55.90	9
QDPAB-C7	10	0.1 M $NaCl/Na_2SO_4$	Cl^{-}/SO_4^{2-}	3.8016	2.475	15.7	10
HPABP-CC3(15)	5	N.A	Cl^{-}/SO_4^{2-}	2.2392	N. A	12.67	11
QP-P3-3			Cl^{-}/SO_4^{2-}	1.728	2.544	5.5	12
QP/P3-4	3.54	0.05 M $NaCl/Na_2SO_4$	Cl^{-}/SO_4^{2-}	1.44	1.8	4.5	12
QP-P11-3			Cl^{-}/SO_4^{2-}	2.7	3.15	8	12
PPO-0.100ImPS	2.5	0.05 M $NaCl/Na_2SO_4$	Cl^{-}/SO_4^{2-}	6.12	4.422	16.12	13
sCOF/aAEM3	10	0.05 M $NaCl/Na_2SO_4$	Cl^{-}/SO_4^{2-}	1.206	0.786	18.92	14
Blend-15 AIEM			Cl^{-}/SO_4^{2-}	1.17	0.87	21.8	15
Blend-10 AIEM			Cl^{-}/SO_4^{2-}	0.864	0.52	14	15
Blend-0 AIEM	2.5	0.05 M $NaCl/Na_2SO_4$	Cl^{-}/SO_4^{2-}	1.17	0.61	11.3	15
PAES-im-2.5c AIEM			Cl^{-}/SO_4^{2-}	1.332	0.765	12.5	16
Neosepta ACS			Cl^{-}/SO_4^{2-}	0.756	0.364	5.27	16
MQ18	N.A	0.01 M $NaCl/Na_2SO_4$	Cl^{-}/SO_4^{2-}	5.22	2.53	16.7	17
AF1-HNN5-50	5.56	0.05 M $NaCl/Na_2SO$	Cl^{-}/SO_4^{2-}	10.512	7.168	62.2	18
PQC76/DSA-0.5			Cl^{-}/SO_4^{2-}	1.8	1.06	10.38	19
PQC76/DSA-0.7	N.A	0.2 M $NaCl/Na_2SO$ and $NaCl/NaNO_3$	Cl^{-}/SO_4^{2-}	1.08	0.58	7.2	19
PQC76/DSA-0.7			Cl^{-}/NO_3^{-}	1.62	0.84	1	19
QA-TB	10	0.1 M $NaCl/Na_2SO_4$	Cl^{-}/SO_4^{2-}	7.3332	N. A	82	20
QPAB-0.5 membrane	10	0.1 M $NaCl/Na_2SO_4$	Cl^{-}/SO_4^{2-}	2.78	1.65	10.5	21
AMX-LPDA#DBSA membranes	8	0.05 M $NaCl/Na_2SO_4$	Cl^{-}/SO_4^{2-}	2.46	2.16	2.13	22
SPPO modified AMX	10	0.1 M $NaCl/Na_2SO_4$	Cl^{-}/SO_4^{2-}	2.844	1.57	52.44	23
QPSF-SF-0.09	5.1	0.05 M $NaCl/Na_2SO_4$	Cl^{-}/SO_4^{2-}	N. A	N. A	15.9	24
AIEM-12C	2.5	0.05 M $NaCl/Na_2SO_4$	Cl^{-}/SO_4^{2-}	0.864	0.52	12.5	25
PBI-O-PhT	3.8	0.014 M $NaCl/NaNO_3/Na_2SO_4$	Cl^{-}/SO_4^{2-}	0.092	-	109	26

Membranes	Current density (mA·cm^{-2})	Feed Concentration	Ion System	Flux (mol m^{-2} h^{-1})	(Normalized flux)	P (Cl$^-$/SO_4^{2-})	Ref
NML-Q_2-NH_2			Cl^-/SO_4^{2-}	1.98	1.98	244.90	
NML-Q_2-NH_2			NO_3^-/SO_4^{2-}	0.86	0.86	84.99	
NML-Q_2-NH_2	5	0.1 M NaCl/Na_2SO_4 and 0.1 M NaCl/Na_2SO_4/$NaNO_3$/NaF/NaBr	Cl^-/NO_3^-	0.45	0.45	0.56	**This work**
NML-Q_2-NH_2			Cl^-/F^-	0.45	0.45	4.15	
NML-Q_2-NH_2			F^-/SO_4^{2-}	0.12	0.12	11.55	

Note S2. Density Functional Theory (DFT) calculations.

DFT calculations were carried out using the exchange correlation functional B3LYP 27, the cc-pvdz basis set 28 and implicit solvation in DMSO using IEFPCM 29, as implemented in Gaussian 16, revision C.01 30. Density and electrostatic potential cube files were generated for the optimized structures using 6 points per Bohr per side in the cube. Electrostatic potential figure heat maps were generated for the range -0.224 to +0.224 a.u.

Note S3. Free Energy calculation of ions

Free energy profiles were obtained from the density profiles of the ions across the polymer-water phase (Figure 3g), using the Boltzmann inversion (Eq (S1)):

$$\frac{\mathbf{\Delta G}}{\mathbf{kT}} = -\ln\left(\frac{[C]}{[C_0]}\right) \qquad \text{(S1)}$$

Where [C] is the position-dependent ion concentration and [C_0] the corresponding ion reference concentration in the bulk pore outside the polymer. The free energy results support the idea that larger and more hydrated ions like SO_4^{2-} are more likely to interact and be retained in the polymer matrix, thus impacting their transport more significantly than smaller ions like Cl^-.

Supplementary References

1. Abraham MJ, Murtola T, Schulz R, Páll S, Smith JC, Hess B, Lindahl E. GROMACS: High performance molecular simulations through multi-level parallelism from laptops to supercomputers. *SoftwareX* **1-2**, 19-25 (2015).

2. Vanommeslaeghe K, Hatcher E, Acharya C, Kundu S, Zhong S, Shim J, Darian E, Guvench O, Lopes P, Vorobyov I, Mackerell Jr AD. CHARMM general force field: A force field for drug-like molecules compatible with the CHARMM all-atom additive biological force fields. *J Comput Chem* **31**, 671-690 (2010).

3. Vanommeslaeghe K, MacKerell AD, Jr. Automation of the CHARMM General Force Field (CGenFF) I: Bond Perception and Atom Typing. *J Chem Inf Model* **52**, 3144-3154 (2012).

4. Riopedre-Fernandez M, Kostal V, Martinek T, Martinez-Seara H, Biriukov D. Developing and Benchmarking Sulfate and Sulfamate Force Field Parameters via Ab Initio Molecular Dynamics Simulations To Accurately Model Glycosaminoglycan Electrostatic Interactions. *Journal of Chemical Information and Modeling* **64**, 7122-7134 (2024).

5. Vanquelef E, Simon S, Marquant G, Garcia E, Klimerak G, Delepine JC, Cieplak P, Dupradeau F-Y. R.E.D. Server: a web service for deriving RESP and ESP charges and building force field libraries for new molecules and molecular fragments. *Nucleic Acids Research* **39**, W511-W517 (2011).

6. Wang F, Becker J-P, Cieplak P, Dupradeau F-Y. RED Python: Object oriented programming for Amber force fields. In: *Abstracts of Papers of the American Chemical Society*). AMER CHEMICAL SOC 1155 16TH ST, NW, WASHINGTON, DC 20036 USA (2014).

7. Tansel B. Significance of thermodynamic and physical characteristics on permeation of ions during membrane separation: Hydrated radius, hydration free energy and viscous effects. *Separation and Purification Technology* **86**, 119-126 (2012).

8. David F, Vokhmin V, Ionova G. Water characteristics depend on the ionic environment. Thermodynamics and modelisation of the aquo ions. *J Mol Liq* **90**, 45-62 (2001).

9. Li J, Xu Z, Liao J, Ang EH, Chen X, Mu J, Shen J. Revolutionary MOF-enhanced anion exchange membrane for precise monovalent anion separation through structural optimization and doping. *Desalination* **576**, 117352 (2024).

10. Yang J, Chen Q, Afsar NU, Ge L, Xu T. Poly(alkyl-biphenyl pyridinium)-Based Anion Exchange Membranes with Alkyl Side Chains Enable High Anion Permselectivity and Monovalent Ion Flux. *Membranes* **13**, 188 (2023).

11. Xu T, Wang Y, Chen Q, Zhu Y, Li W, Sheng F, Ge L, Li X, Xu T. Scalable and interfacial gap-free mixed matrix membranes for efficient anion separation. *AIChE J* **70**, e18242 (2024).

12. Irfan M, Ge L, Wang Y, Yang Z, Xu T. Hydrophobic Side Chains Impart Anion Exchange Membranes with High Monovalent–Divalent Anion Selectivity in Electrodialysis. *ACS Sustainable Chem Eng* **7**, 4429-4442 (2019).

13. Li J, Qian H, Liao J, Li Y, Xu J, Chen Q, Yao Y, Mu J, Xu Y, Ruan H, Xu X, Shen J. Enhanced monovalent anion selectivity of poly(2,6-dimethyl-1,4-phenylene oxide)-based amphoteric ion exchange membranes having rough surface. *Journal of Membrane Science* **661**, 120911 (2022).

14. Wang Y, Ren L, Wang J, Zhao J, Chen Q-B. In-situ growth of anionic covalent organic frameworks efficaciously enhanced the monovalent selectivity of anion exchange membranes. *Journal of Membrane Science* **659**, 120818 (2022).

15. Liao J, Chen Q, Pan N, Yu X, Gao X, Shen J, Gao C. Amphoteric blend ion-exchange membranes for separating monovalent and bivalent anions in electrodialysis. *Separation and Purification Technology* **242**, 116793 (2020).

16. Liao J, Yu X, Pan N, Li J, Shen J, Gao C. Amphoteric ion-exchange membranes with superior mono-/bi-valent anion separation performance for electrodialysis applications. *Journal of Membrane Science* **577**, 153-164 (2019).

17. Mondal R, Sarkar S, Patnaik P, Chatterjee U. Preparation of a Monovalent-Selective Anion-Exchange Membrane: Effect of Alkyl Chain Length and Crystallinity. *ACS Applied Polymer Materials* **5**, 2513-2524 (2023).

18. Gangrade AS, Tusi B, Ghosh PC, Holdcroft S. High monovalent/divalent permselectivity and low ionic resistance of ionene-based anion exchange membranes in electrodialysis. *Journal of Membrane Science* **685**, 121906 (2023).

19. Zhang H, Ding R, Zhang Y, Shi B, Wang J, Liu J. Stably coating loose and electronegative thin layer on anion exchange membrane for efficient and selective monovalent anion transfer. *Desalination* **410**, 55-65 (2017).

20. Liu M-L, Chen Y, Hu C, Zhang C-X, Fu Z-J, Xu Z, Lee YM, Sun S-P. Microporous membrane with ionized sub-nanochannels enabling highly selective monovalent and divalent anion separation. *Nature Communications* **15**, 7271 (2024).

21. Chen Q, Zhu Y, Jiang C, Song W, Ye Q, He H, Ding Z, Fu R, Liu Z, Ge L, Xu T. Pilot-scale poly(alkyl-biphenyl pyridine)-based monovalent anion perm-selective

membranes with exceptional Cl−/SO42− selectivity in ion-distillation. *Journal of Membrane Science* **702**, 122787 (2024).

22. Lejarazu-Larrañaga A, Zhao Y, Molina S, García-Calvo E, Van der Bruggen B. Alternating current enhanced deposition of a monovalent selective coating for anion exchange membranes with antifouling properties. *Separation and Purification Technology* **229**, 115807 (2019).

23. Jiang C, Zhang D, Muhammad AS, Hossain MM, Ge Z, He Y, Feng H, Xu T. Fouling deposition as an effective approach for preparing monovalent selective membranes. *Journal of Membrane Science* **580**, 327-335 (2019).

24. Pan J, Ding J, Zheng Y, Gao C, Van der Bruggen B, Shen J. One-pot approach to prepare internally cross-linked monovalent selective anion exchange membranes. *Journal of Membrane Science* **553**, 43-53 (2018).

25. Li J, Chen X, Liao J, Li Y, Mu J, Xu Y, Du Y, Ruan H, Xu X, Shen J. The endowment of monovalent anion selectivity and antifouling property to cross-linked ion-exchange membranes by constructing amphoteric structure. *Separation and Purification Technology* **328**, 125104 (2024).

26. Lysova AA, Manin AD, Golubenko DV, Ponomarev II, Altynov VA, Hilal N, Yaroslavtsev AB. Ultra-high nitrate-selective metal-polymer membranes based on cardo polybenzimidazole for electrodialysis. *Journal of Membrane Science* **716**, 123518 (2025).

27. Becke AD. Density‐functional thermochemistry. I. The effect of the exchange‐only gradient correction. *The Journal of Chemical Physics* **96**, 2155-2160 (1992).

28. Dunning TH, Jr. Gaussian basis sets for use in correlated molecular calculations. I. The atoms boron through neon and hydrogen. *The Journal of Chemical Physics* **90**, 1007-1023 (1989).

29. Scalmani G, Frisch MJ. Continuous surface charge polarizable continuum models of solvation. I. General formalism. *The Journal of Chemical Physics* **132**, 114110 (2010).

30. Frisch M, Trucks G, Schlegel H, Scuseria G, Robb M, Cheeseman J, Scalmani G, Barone V, Petersson G, Nakatsuji HJGIWC. Gaussian 16 Revision C. 01, 2016. **1**, 572 (2016).
